# Comparative analysis of differentially expressed genes between the ovaries from pregnant and nonpregnant goats using RNA-Seq

**DOI:** 10.1186/s40709-019-0095-9

**Published:** 2019-05-06

**Authors:** Qing Quan, Qi Zheng, Yinghui Ling, Fugui Fang, Mingxing Chu, Xiaorong Zhang, Yong Liu, Wenyong Li

**Affiliations:** 1 College of Animal Science and Technology, Anhui Agricultural University, Hefei, 230036 Anhui China; 2 Local Animal Genetic Resources Conservation and Biobreeding Laboratory of Anhui Province, Hefei, 230036 Anhui China; 3 College of Economy and Technology, Anhui Agricultural University, Hefei, 230036 Anhui China; 4 Key Laboratory of Farm Animal Genetic Resources and Germplasm Innovation of Ministry of Agriculture, CAAS, Beijing, 100193 China; 5 Key Laboratory of Embryo Development and Reproductive Regulation of Anhui Province, Fuyang Normal University, Fuyang, Anhui 236037 China

**Keywords:** Differentially expressed genes, Ovary, Pregnant and nonpregnant, Goat, RNA-Seq

## Abstract

**Background:**

A multitude of genes tightly regulate ovarian follicular development and hormone secretion. These complex and coordinated biological processes are altered during pregnancy. In order to further understand the regulatory role of these genes during pregnancy, it is important to screen the differentially expressed genes (DEGs) in the ovaries of pregnant and nonpregnant mammals. To detect the genes associated with the development of pregnancy in goats, DEGs from the ovaries from pregnant and nonpregnant Anhui white goats (pAWGs and nAWGs, respectively) were analyzed using RNA sequencing technology (RNA-Seq).

**Results:**

In this study, 13,676,394 and 13,549,560 clean reads were generated from pAWGs and nAWGs, respectively, and 1724 DEGs were identified between the two libraries. Compared with nAWGs, 1033 genes were upregulated and 691 genes were downregulated in pAWGs, including *PGR*, *PRLR*, *STAR* and *CYP19A1*, which play important roles in goat reproduction. Gene Ontology analysis showed that the DEGs were enriched for 49 functional GO terms. Kyoto Encyclopedia of Genes and Genomes analysis revealed that 397 DEGs were significantly enriched in 13 pathways, including “cell cycle”, “cytokine–cytokine receptor interaction” and “steroid biosynthesis”, suggesting that the genes may be associated with cell cycle regulation, follicular development and hormone secretion to regulate the reproduction process. Additionally, quantitative real-time PCR was used to verify the reliability of the RNA-Seq data.

**Conclusions:**

The data obtained in this work enrich the genetic resources of goat and provide a further understanding of the complex molecular regulatory mechanisms occurring during the development of pregnancy and reproduction in goats. The DEGs screened in this study may play an important role in follicular development and hormone secretion and they would provide scientific basis for related research in the future.

**Electronic supplementary material:**

The online version of this article (10.1186/s40709-019-0095-9) contains supplementary material, which is available to authorized users.

## Background

The goat (*Capra hircus*) can provide high-quality wool, meat, and other products and it is thus an important domestic and commercial animal. However, compared with other commercial animals, goat fecundity is relatively low, which has hindered the development of the goat industry. Anhui white goats (AWGs) have been used as a model to study goat fecundity in recent years [1] because of its precocious puberty, higher fertility, and higher leather quality than other types of goats [2].

Pregnancy is one of the most complex reproductive activities of mammals and is tightly regulated by the external environment, various endocrine factors, and the expression of a large number of genes [3, 4]. The ovary plays a vital role during pregnancy. There are significant differences in the activity and endocrine characteristics of the ovary during pregnancy and nonpregnancy [5]. In the nonpregnant phase, ovulation is normal and estrogen secretion dominates to maintain normal estrous function; in contrast, with the formation of the corpus luteum, ovulation is suspended during pregnancy and progesterone secretion is gradually increased to maintain pregnancy [6]. These differences between pregnancy and nonpregnancy may be associated with the distinct regulation of gene expression, which is implicated in various reproductive activities, including follicular development [7, 8], hormone secretion [9], ovulation [10], luteinization [11], and pregnancy maintenance [12].

Many studies have indicated that the differential expression of ovarian mRNA hormone receptor genes (follicle-stimulating hormone receptor [*FSHR*] and luteinizing hormone/choriogonadotropin receptor [*LHR*]) and growth factor genes (insulin-like growth factor 1 [*IGF*-*I*] and bone morphogenetic protein 6 [*BMP6*]) during follicular development significantly impact the development of preantral follicles [13–15]. Pramod et al. showed that the differential expression of bone morphogenetic protein 15 (*BMP*-*15*) and growth/differentiation factor (*GDF*-*9*) might be involved in higher prolificacy in goats [16]. Suppression subtractive hybridization has been used to screen differentially expressed genes (DEGs) in ovarian tissues between polytocous and monotocous goats, obtaining 29 differentially expressed sequence tags [17]. Microarray analysis has been used to analyze gene expression during early folliculogenesis in goats, and 2466 genes and three cell death-related pathways were explored [18]. In 2014, the different expression of genes in the ovaries from uniparous and multiparous AWGs were analyzed by high-throughput sequencing. In total, 2201 DEGs were identified, and 12 genes that may be associated with AWG high prolificacy were explored [19]. However, the information on the functions of specific genes in regulating goat pregnancy and reproduction is insufficient.

RNA sequencing technology (RNA-Seq) is mainly used for quantitative gene expression analysis of biological processes in a particular tissue or cells in specific species. With the development of high-throughput technologies, RNA-Seq has been extensively used to study specific genes in numerous species, including mice, pig, cattle, human, sheep, and goat [19–24]. It was reported that 338 genes were upregulated in the Jining grey goats and 404 were upregulated in Laiwu blank goats, and these genes might be involved in the regulation of goat fecundity and prolificacy [25]. Ling et al. used RNA-Seq to study the DEGs between the ovaries of uniparous and multiparous goats, and identified candidate genes for goat prolificacy [19].

In the present study, we analyzed the gene expression levels in ovaries from pregnant and nonpregnant AWGs (pAWGs and nAWGs, respectively) using an Ion Proton™ RNA-Seq platform. The results shed further light on the role of specific genes in reproductive biological processes, including hormone secretion, ovulation, luteinization formation, and pregnancy maintenance, which will help to identify genes that could potentially be used to regulate hircine reproduction and breeding practices in the future.

## Methods

### Animal materials

In the present study, AWGs raised under identical feeding conditions at the College of Animal Science and Technology (Anhui Agricultural University, Hefei, China) were used as animal samples. Six ewes, which were 2 years old and had a similar appearance, were selected from two groups for this study. Three of them had been pregnant for 3 months, and the rest were not pregnant and were undergoing anestrus, which was determined by hormone levels. After slaughter, both whole ovaries from each ewe were immediately collected and frozen in liquid nitrogen, followed by storage at − 80 °C. Half of the ovaries were used for RNA-Seq, and the other half were used for real-time PCR. All experimental procedures involving AWGs had been approved by the ethics committee of Anhui Agricultural University, Anhui, China (permit no. AHAU20101025).

### Total RNA extraction and analysis for RNA-Seq

To minimize differences among individuals, one ovary from each pair of ovaries from the three nAWGs and three pAWGs were grouped for RNA extraction using RNAiso Plus (Takara, Dalian, China) following the manufacturer’s protocol. Briefly, the correct amount of frozen tissue sample was dissolved in 1 ml RNAiso Plus. After incubation at room temperature for 5 min, the homogenate was centrifuged at 12,000*g* for 5 min at 4 °C. The supernatant was then transferred to a new centrifuge tube. Chloroform (1/5 volume of RNAiso Plus) was then added to the supernatant. The solution was fully emulsified and incubated for 5 min at room temperature before centrifugation at 12,000*g* for 15 min at 4 °C. The supernatant was then carefully transferred to another centrifuge tube. An equal amount of isopropanol was added to the supernatant. The solution was fully mixed and incubated for 10 min at room temperature before an additional centrifugation at 12,000*g* for 10 min at 4 °C. Then, 1 ml of 75% ethanol was slowly added to the white precipitate, which was centrifuged at 12,000*g* for 5 min at 4 °C. Finally, the supernatant was discarded, and the precipitate was dried and dissolved in RNase-free ddH_2_O. The quantity and quality of the total RNA were measured using the Agilent 2100 Bioanalyzer system (Santa Clara, CA), and the samples were stored at − 80 °C until analysis.

### Library preparation and sequencing

Two groups of total RNA were used for library preparation and sequencing by pooling equal quantities (10 μg) of total RNA isolated from 3 individual pregnant or 3 individual nonpregnant goat ovaries. Briefly, the total RNA samples were treated with RNase-free DNase I (Ambion Inc., Austin, TX) to degrade any possible genomic DNA contamination. The digested products were then purified by magnetic beads. Next, the mRNA was enriched using oligo (dT) magnetic beads (for eukaryotes) and fragmented into short fragments (approximately 200 bp) using fragmentation buffer. First-strand cDNA was then synthesized with random hexamer primers using the mRNA fragments as templates. Buffer, dNTPs, RNase H, and DNA polymerase I were added to synthesize the second strand cDNA. The double-stranded cDNA was purified with the QIAQuick PCR extraction kit (Qiagen, Germany) and eluted with elution buffer for end repair and poly (A) addition. Sequencing adapters were then ligated to the 5′ and 3′ ends of the fragments. Next, the ligation products were size selected and purified by agarose gel electrophoresis. Finally, the fragments were enriched by PCR amplification (95 °C for 10 min, followed by 40 cycles at 95 °C for 15 s and 60 °C for 45 s), purified by magnetic beads, and dissolved in the appropriate amount of elution buffer. During the quality control steps, an Agilent 2100 Bioanalyzer (Agilent, USA) was used for qualification and quantification of the sample library. Finally, the cDNA libraries were sequenced using an Ion Proton platform (Life Technologies, USA) at the Beijing Genomics Institute (BGI) in Shenzhen, China.

### Sequencing data analysis

According to the requirements of this experiment and the principles of standard bioinformatics analysis, some contaminant reads, such as low-quality reads, adaptor sequences and reads with a length less than the threshold (30 bp), should be removed from the raw reads [26]. After filtering, the valid sequences, also known as the clean reads, were retained for further analysis. To identify the gene expression patterns in each genotype from the two libraries, the clean reads were mapped to goat reference gene sequences (http://goat.kiz.ac.cn/GGD/download.htm) and goat reference genome sequences (http://goat.kiz.ac.cn/GGD/download.htm) using SOAPaligner/SOAP2 [27]. No more than two mismatches were allowed in the alignment.

### Identification of DEGs

To identify the DEGs between pAWGs and nAWGs, the gene expression levels were calculated using the reads per kilobase per million reads (RPKM) as follows [20]:

$$RPKM(A) = \frac{{10^{6} C}}{{{\raise0.7ex\hbox{${NL}$} \!\mathord{\left/ {\vphantom {{NL} {10^{3} }}}\right.\kern-0pt} \!\lower0.7ex\hbox{${10^{3} }$}}}}$$

where RPKM (A) is the expression level of gene A, C is the number of reads uniquely aligning to gene A, N is the total number of reads uniquely aligning to all genes, and L is the number of bases in gene A. DEGs were identified using a strict algorithm developed by BGI based on the method described by Audic and Claverie [28]. To compare the differential expression of the two samples, fold changes and *p* values were used, which calculated from the normalized expression using the following formulas:

$${\text{Fold-change}} = \log_{2} \left( {{\text{RPKM}}\;{\text{of}}\;{\text{Pergnant}}\;{\text{Ovary/RPKM}}\;{\text{of}}\;{\text{Nonpregnant}}\;{\text{Ovary}}} \right)$$

Formula for the *p* value:

$$2\sum\limits_{i = 0}^{{i = {\text{y}}}} {p\left( {i\left| x \right.} \right)} \;or\;2 \times \left( {1 - \sum\limits_{i = o}^{i = y} {p\left( {i\left| x \right.} \right)} } \right)\;\left( {if\sum\limits_{i = 0}^{i = y} {p\left( {i\left| x \right.} \right) > 0.5} } \right)$$

$$p\left( {y\left| x \right.} \right) = \left( {\frac{{N_{2} }}{{N_{1} }}} \right)^{y} \frac{{\left( {x + y} \right)!}}{{x!y!\left( {1 + \frac{{N_{2} }}{{N_{1} }}} \right)^{{\left( {x + y + 1} \right)}} }}$$

N_1_ and x represent the total clean reads count and the normalized expression level of a given gene in the nonpregnant ovary library, respectively. N_2_ and y represent the total clean reads count and the normalized expression level of a given gene in the pregnant ovary library, respectively. The false discovery rate (FDR) was used to determine the threshold *p* values in the multiple tests and perform analyses through the manipulation of the FDR values. In the present study, FDR ≤ 0.001 and |log_2_Ratio| ≥ 1 were used as the thresholds to judge the significance of the gene expression differences [29].

### Real-time quantitative PCR

Eight DEGs were randomly selected to validate the RNA-Seq (quantification) data through real-time quantitative PCR (qPCR). The primers were designed using the Primer Express 5.0 software (ABI, China) (Table 1). GAPDH was used as a reference gene for data standardization. The left three ovaries from each nAWG or pAWG were pooled for total RNA extraction as described above. First-strand cDNA was synthesized using 1 μg of total RNA and the First-Strand cDNA Synthesis Kit (Toyobo, Japan). The qPCR reactions were performed on a Step One Plus™ Real-Time PCR System (Life Technologies, USA) using THUNDERBIRD SYBR qPCR Mix (Toyobo, Japan). The reaction contained 2.0 μl cDNA, 12.5 μl qPCR Mix, 2.0 μl of each primer, and 8.5 μl RNase-free H_2_O. The thermal cycling conditions were 95 °C for 1 min, followed by 40 cycles of 95 °C for 15 s, 58 °C for 20 s, and 72 °C for 20 s. All reactions were performed in triplicate. Relative quantification analyses were performed using the comparative CT method, and the relative gene expression levels were calculated using the 2^−ΔΔCT^ method [30]. All data are expressed as the mean ± standard deviation and were analyzed by Student’s t-test with SPSS 17.0 for Windows (SPSS Inc. Released 2008. SPSS Statistics for Windows, Version 17.0. SPSS Inc, Chicago, USA). Data were considered statistically significant at *p* < 0.05.

**Table 1:** The primers used for real-time quantitative PCR analysis

Gene name	Primer sequence
*SST*	S: 5′-ATCCCCGACTCCGTCAGTTT-3′ A: 5′-GCTCCAGCCTCATTTCATCCT-3′
*SLC1A2*	S: 5′-AAATGAATGGCGTCGTCCTAG-3′ A: 5′-GCCGTCAGGATAAGGAGCAT-3′
*TSPAN1*	S: 5′-GGCTCGCCCTATGTAAGAATG-3′ A: 5′-TGACTGCGTTGGTTCGGAT-3′
*OSTF1*	S: 5′-AAGCAACTATGTGGCAGAGCAG-3′ A: 5′-AAGCCCAGTAGAGGGCAGTG-3′
*DEFB112*	S: 5′-TGCCCAACTCACAACAGAACAT-3′ A: 5′-TGGTGGCACAGAAATTCCAAC-3′
*UGDH*	S: 5′-CCCTATGAAGCCTGTGATGGT-3′ A: 5′-CCGTCAAAGATAAAGGCTGGT-3′
*APOA2*	S: 5′-CTTGCATTGACTGTGCTGCTC-3′ A: 5′-TCCATGAGGTCCTTGCCATAG-3′
*FADS1*	S: 5′-TCCGCAAAGACCCTGACATC-3′ A: 5′-ATTTGTGCTGGTAGTTGTAGGGC-3′
*GAPDH*	S: 5′-GCATCGTGGAGGGACTTATGAC-3′ A: 5′-CGGCAGGTCAGATCCACAAC-3′

### GO function enrichment and KEGG pathway analysis of the DEGs

Gene Ontology (GO) enrichment analysis provided all the GO terms that were significantly enriched for the DEGs compared with the genome background and filtered the DEGs that correspond to biological functions. First, the DEGs were mapped to the GO terms in the database (http://www.geneontology.org/), and the gene numbers for each term were calculated. A hypergeometric test was then used to find significantly enriched GO terms among the DEGs compared with the genome background. The formula for the calculation is as follows:

$$p = 1 - \sum\limits_{i = 0}^{m - 1} {\frac{{\left( {\begin{array}{*{20}c} M \\ i \\ \end{array} } \right)\left( {\begin{array}{*{20}c} {N - M} \\ {n - i} \\ \end{array} } \right)}}{{\left( {\begin{array}{*{20}c} N \\ n \\ \end{array} } \right)}}}$$

where N is the number of genes with GO annotations, n is the number of DEGs in N, M is the number of genes that are annotated to specific GO terms, and m is the number of DEGs in M. The calculated *p* value underwent Bonferroni correction using a corrected *p* value ≤ 0.05 as a threshold. GO terms fulfilling this condition were defined as significantly enriched GO terms for the DEGs. This analysis is able to recognize the main biological functions performed by the DEGs.

Using nr annotation, the gene annotation of the DEGs was conducted using Blast2GO [31]. After obtaining the GO annotation for the DEGs, Web Gene Ontology Annotation Plot (WEGO) software was used to perform GO functional classification of the DEGs and understand the distribution of the gene functions from the species at the macro level [32].

The Kyoto Encyclopedia of Genes and Genomes (KEGG) is the major public pathway-related database [33]. Pathway enrichment analysis identified significantly enriched metabolic pathways or signal transduction pathways for the DEGs compared with the whole genome background. The formula for calculating the pathways is the same as for the GO analysis, with N as the number of all genes with KEGG annotations, n as the number of DEGs in N, M as the number of genes annotated to specific pathways, and m as the number of DEGs in M. Pathways with Q values ≤ 0.05 are significantly enriched for the DEGs.

## Results

### Overview of sequencing data

**Fig. 1:**
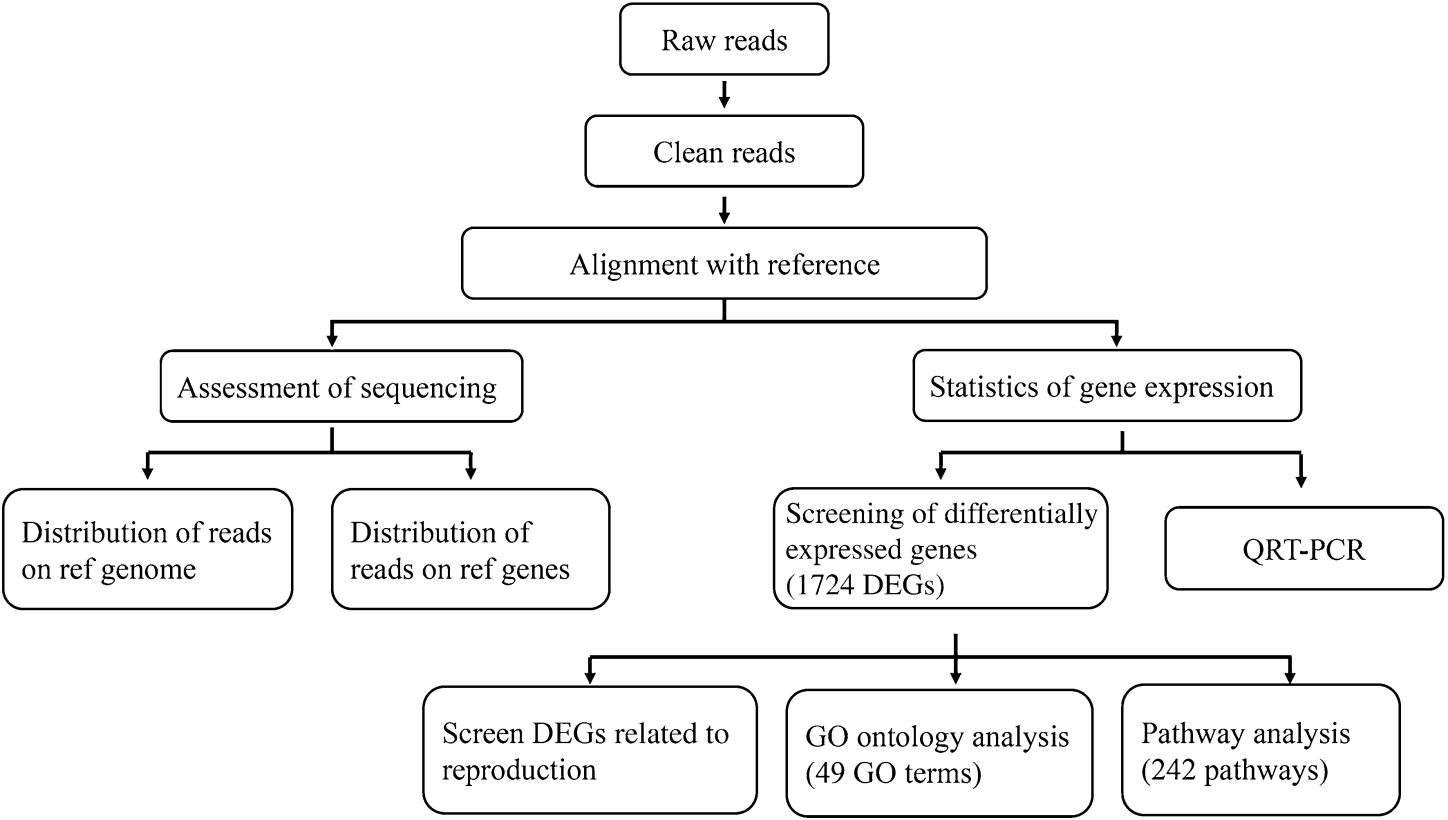
Summary of genetic analysis procedures for pregnant and unpregnant goats

In this study, we generated cDNA libraries of ovaries from pregnant and nonpregnant goat (*Capra hircus*) (Fig. 1). Approximately 14.39 Gb reads, including 7.12 Gb from pAWGs and 7.27 Gb from nAWGs, were obtained through the sequencing of two ovary cDNA libraries from pAWGs and nAWGs using the Ion Proton sequencing platform at BGI in Shenzhen, China. After discarding sequences shorter than 30 bp, filtering the adaptor sequences, and eliminating low-quality sequences from the raw data, 13,676,394 and 13,549,560 clean reads were ultimately obtained from the ovary libraries for pAWGs and nAWGs, respectively, and were retained for further analysis. The clean reads from each library were sufficient for the quantitative analysis of gene expression.

According to the mapping analysis, approximately 98% of the reads (98.01% for nAWGs and 97.95% for pAWGs) could be mapped to the goat genome sequence. When the unique matches were regarded, slightly more than 92% (92.50% for nAWGs and 92.72% for pAWGs) of the reads corresponded to the genome, and approximately 45% of the reads (45.03% for nAWGs and 43.47% for pAWGs) could be perfectly matched to the genome.

When the reference genes were used to analyze the data, only 75.91% of the reads in nAWGs and 75.64% of the reads in pAWGs could be mapped to reference genes. Regarding the unique matches, 54.14% of the reads in nAWGs and the 54.54% in pAWGs corresponded to the reference genes. Additionally, 37.83% of the reads in nAWGs and 36.43% of the reads in pAWGs could be perfectly matched to the reference genes. The major characteristics of the two libraries are described in Additional file 1.

### Identification and analysis of the DEGs in the ovaries from pregnant and nonpregnant goats

In total, 20,651 genes were detected in the study, among which 17,181 genes were co-expressed in the two libraries and 2002 and 1468 genes were specifically expressed in the ovary libraries from pAWGs and nAWGs, respectively (Additional file 2).

Analysis of the gene coverage distribution indicated that 16% of genes had 70–90% coverage, while 26% of genes in the nAWG and 27% of genes in the pAWG libraries had 90–100% coverage (Additional file 3: Fig. S1), which means the read distributions were similar between the two libraries.

To detect pregnancy-related genes, the expression levels of all the detected genes from pAWGs and nAWGs were calculated using the RPKM method. An FDR ≤ 0.001 and the absolute value of the log_2_ ratio ≥ 1 were used as criteria to judge the significant differences in the expressed genes. A total of 1724 DEGs were detected between the nAWG and pAWG libraries, with 1033 upregulated genes and 691 downregulated genes in pAWGs compared to nAWGs (Additional file 4: Fig. S2 and Additional file 5). Some of the DEGs were related to reproduction and reproductive processes, such as progesterone receptor (*PGR*), prolactin receptor precursor (*PRLR*), steroidogenic acute regulatory protein (*STAR*), cytochrome P450 19A1 (*CYP19A1*) and so on (Table 2). Among the DEGs, 15 genes and 71 genes were found to be specifically expressed in the nAWG and pAWG libraries, respectively. Among the specifically expressed DEGs, we screened four DEGs related to reproduction, including two genes [beta-defensin 112 (*DEFB112*) and interferon epsilon (*IFNE*)] that were specifically expressed in nAWGs and two genes [somatostatin precursor (*SST*) and somatostatin receptor type 1 (*SSTR1*)] that were specifically expressed in pAWGs.

**Table 2:** Details of the differentially expressed genes (DEGs) associated with reproduction

Gene ID	Description	log_2_ ratio (pregnant ovary/non-pregnant ovary)	*p* value
gi|548512269|ref|XM_005694809.1	gi|339460411|gb|AEJ76924.1|/0/prolactin receptor long form	9.971617	1.46E−4
gi|548524371|ref|XM_005698829.1	gi|57163979|ref|NP_001009243.1|/5.24994e-157/steroidogenic acute regulatory protein, mitochondrial precursor	6.621061	4.65E−345
gi|548458406|ref|XM_005677785.1	gi|440906469|gb|ELR56725.1|/0/3 beta-hydroxysteroid dehydrogenase/Delta 5–>4-isomerase, partial	5.578285	3.93E−398
gi|548485096|ref|XM_005686390.1	gi|426223863|ref|XP_004006093.1|/1.84864e-105/PREDICTED:3-oxo-5-alpha-steroid 4-dehydrogenase 2	4.690717	8.63E−14
gi|548470003|ref|XM_005681450.1	gi|162946588|gb|ABY21280.1|/4.77201e-131/osteopontin	4.535885	1.66E−367
gi|548485794|ref|XM_005686598.1	gi|426226402|ref|XP_004007333.1|/0/PREDICTED:lutropin-choriogonadotropic hormone receptor	3.872696	8.91E−327
gi|548469920|ref|XM_005681410.1	gi|193876570|gb|ACF24869.1|/0/bone morphogenetic protein 1B	3.614874	9.33E−19
gi|548503561|ref|XM_005692140.1	gi|1911705|gb|AAB50810.1|/0/11 beta-hydroxysteroid dehydrogenase type 2	3.415309	1.40E−40
gi|548500629|ref|XM_005691343.1	gi|426247178|ref|XP_004017363.1|/0/PREDICTED: scavenger receptor class B member 1	3.208502	1.10E−288
gi|548472488|ref|XM_005682375.1	gi|426228997|ref|XP_004008580.1|/0/PREDICTED: low-density lipoprotein receptor	1.829713	2.89E−384
gi|548476777|ref|XM_005683765.1	gi|426222344|ref|XP_004005354.1|/0/PREDICTED: very low-density lipoprotein receptor	1.749974	1.38E−44
gi|548456104|ref|XM_005677120.	gi|426217043|ref|XP_004002763.1|/0/PREDICTED: 3-keto-steroid reductase	1.467141	4.31E−19
gi|548518653|ref|XM_005696914.1	gi|296473962|tpg|DAA16077.1|/1.29128e-162/TPA: bone morphogenetic protein 6-like	1.434549	3.27E−30
gi|548517737|ref|XM_005696509.1	gi|254892264|dbj|BAH86639.1|/2.61573e-94/vascular endothelial growth factor 188	1.225192	2.47E−70
gi|548496483|ref|XM_005690127.1	gi|426245339|ref|XP_004016470.1|/4.26298e-173/PREDICTED:estradiol 17-beta-dehydrogenase 12	1.071481	8.17E−25
gi|548454974|ref|XM_005676599.1	gi|347990733|gb|AEP40507.1|/1.39972e-120/INHA	− 5.33697	1.21E−322
gi|548463672|ref|XM_005679345.1	gi|338807808|gb|AEJ07667.1|/0/inhibin beta A subunit	− 4.78646	2.89E−311
gi|548483458|ref|XM_005685776.1	gi|172088165|ref|NP_001116472.1|/0/cytochrome P450 19A1	− 3.42394	6.98E−348
gi|548454408|ref|XM_005676322.1	gi|440900738|gb|ELR51808.1|/1.70145e-143/Inhibin beta B chain, partial	− 2.67258	7.97E−227
gi|548510550|ref|XM_005694322.1	gi|440903234|gb|ELR53921.1|/1.18142e-66/Estradiol 17-beta-dehydrogenase 1	− 2.52176	2.49E−96
gi|548485697|ref|XM_005686583.1	gi|193876578|gb|ACF24872.1|/0/follicle stimulating hormone receptor	− 2.22307	1.24E−12
gi|548483859|ref|XM_005685967.1	gi|193876584|gb|ACF24875.1|/0/estrogen receptor beta	− 1.62217	6.99E−11
gi|548494087|ref|XM_005689375.1	gi|426245562|ref|XP_004016579.1|/0/PREDICTED:progesterone receptor	− 1.27381	7.88E−15

### QPCR validation

**Fig. 2:**
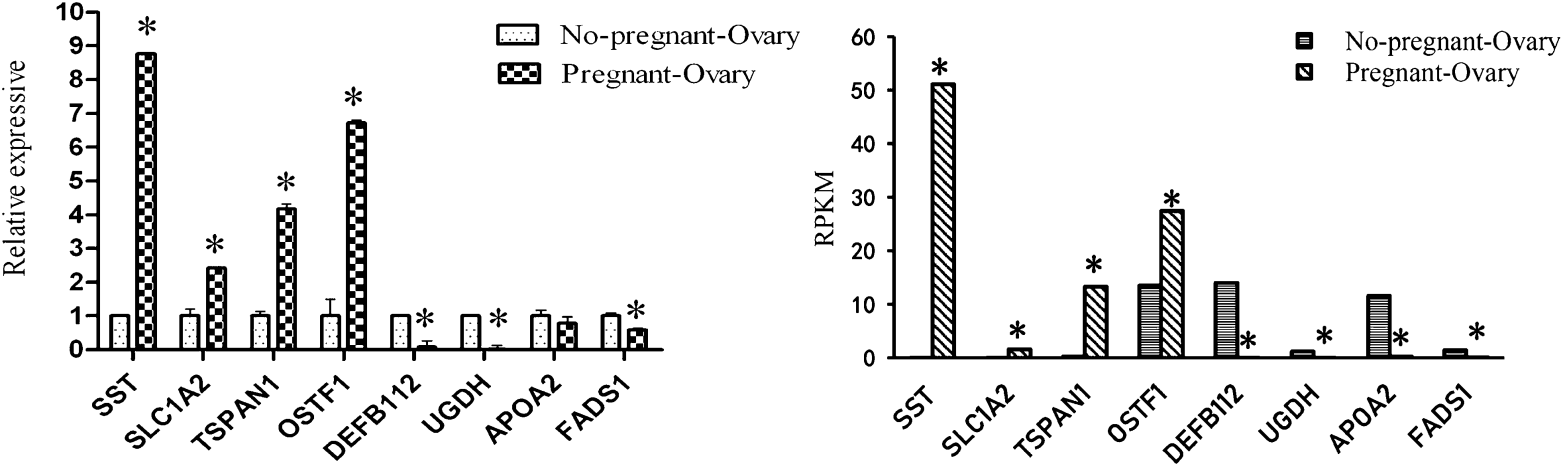
Comparison of RNA-Seq (Quantification) and qPCR results. Results represent the mean (± SD) of three experiments. *p ≤ 0.05

The expression profiles of eight randomly selected DEGs were validated using qPCR analysis. As shown in Fig. 2, the expression of *SST*, *SLC1A2*, *TSPAN1*, and *OSTF1* was upregulated, whereas the expression of *DEFB112*, *UGDH*, *APOA2*, and *FADS1* was downregulated in pAWGs compared with nAWGs. These expression patterns were largely consistent with the RNA-Seq (Quantification) results, which means that the RNA-Seq results were reliable and that RNA-Seq could be used to reliably and accurately perform mRNA differential expression analysis.

### GO functional enrichment and KEGG pathway analysis of the DEGs

**Fig. 3:**
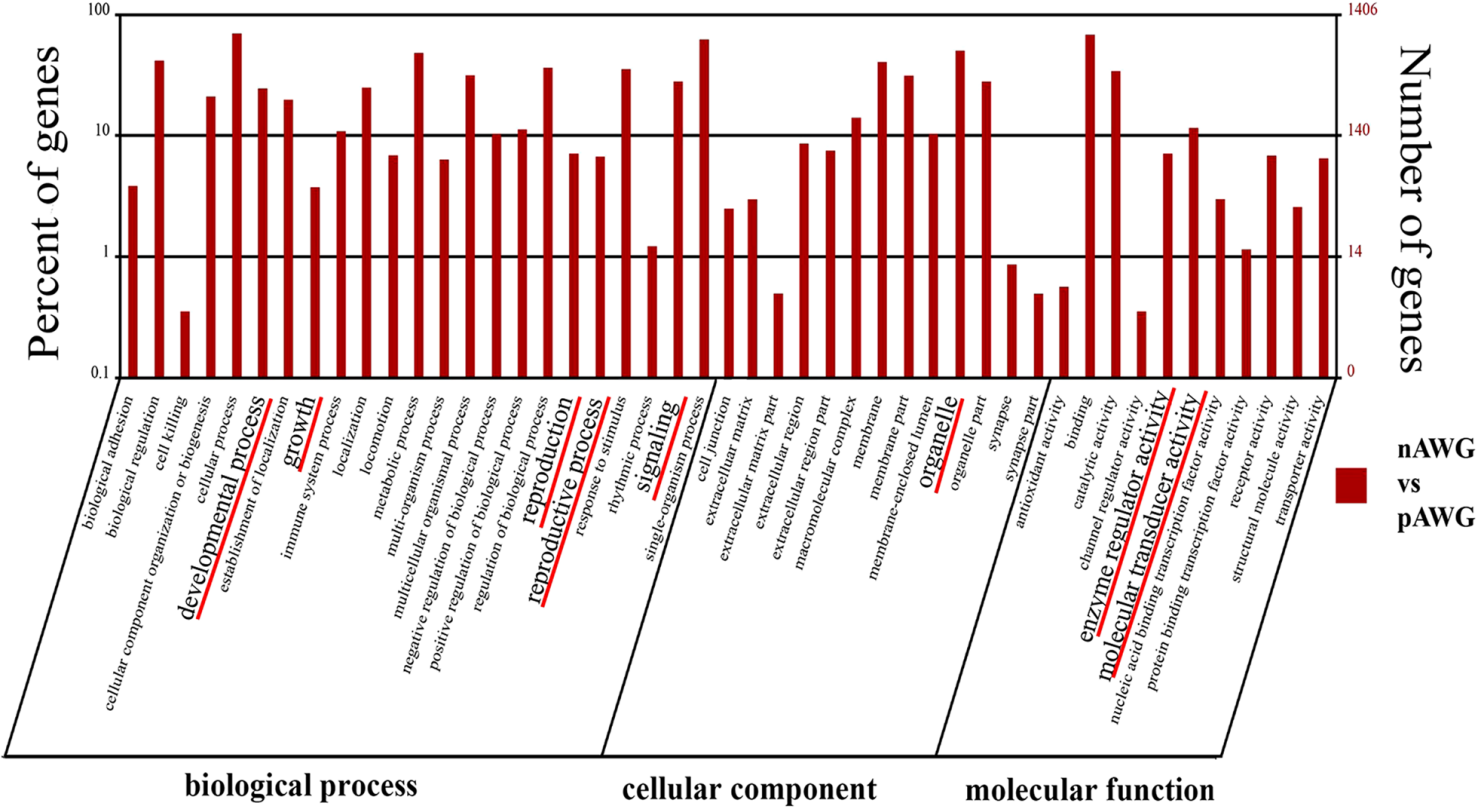
Histogram of the Gene Ontology (GO) classifications. The results are summarized for the following three main categories: “biological process”, “cellular component”, and “molecular function”. Among these groups, the GO terms “cellular process”, “binding”, “cell”, and “cell part” predominated in each of the three main categories. The reproduction-related GO terms were underlined

GO is an international standardized classification system for gene function that supplies a set of controlled terms to comprehensively describe the properties of genes and gene products. In our present study, GO function enrichment was used to screen the DEGs that were significantly associated with different biological functions. The well-annotated gene sequences were assigned to 23, 15, and 11 functional groups in the “biological process”, “cellular component”, and “molecular function” categories, respectively (Additional file 6). Many of the enriched GO terms are reproduction-related, such as “signaling”, “reproduction”, “reproduction process”, “developmental process”, “growth”, “organelle”, “receptor activity”, “biological regulation”, “regulation of biological process”, “molecular transducer activity” and “enzyme regulator activity” (Fig. 3). These GO terms were mostly associated with cell proliferation, apoptosis, follicular development, hormone secretion, and pregnancy maintenance. Among the reproduction-related GO terms, some important genes related to reproduction were detected, including *PGR*, *CYP19A1*, *CCNB1*, *STAR*, *SST*, *SSTR1*, estrogen receptor beta 2 (*ESR2*), BMP receptor 1B (*BMPR1B*) and erythropoietin receptor (*EPOR*).

**Fig. 4:**
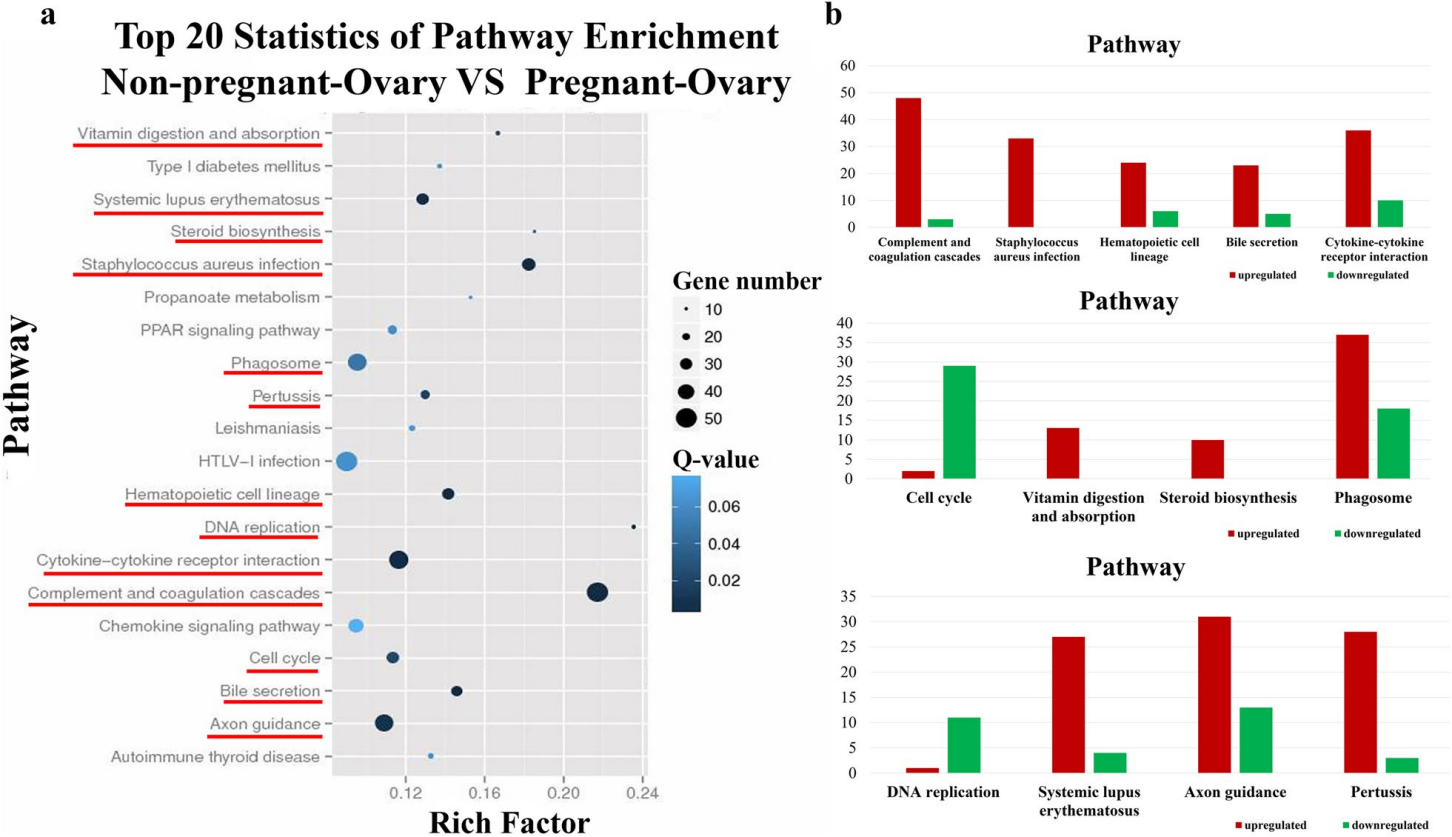
Top 20 pathway enrichment assignments for nonpregnant and pregnant ovaries. The Q value is the corrected *p* value ranging from 0 to 1. The lower the Q value, the greater the intensity. Red lines are significantly enriched signal pathways

**Fig. 5:**
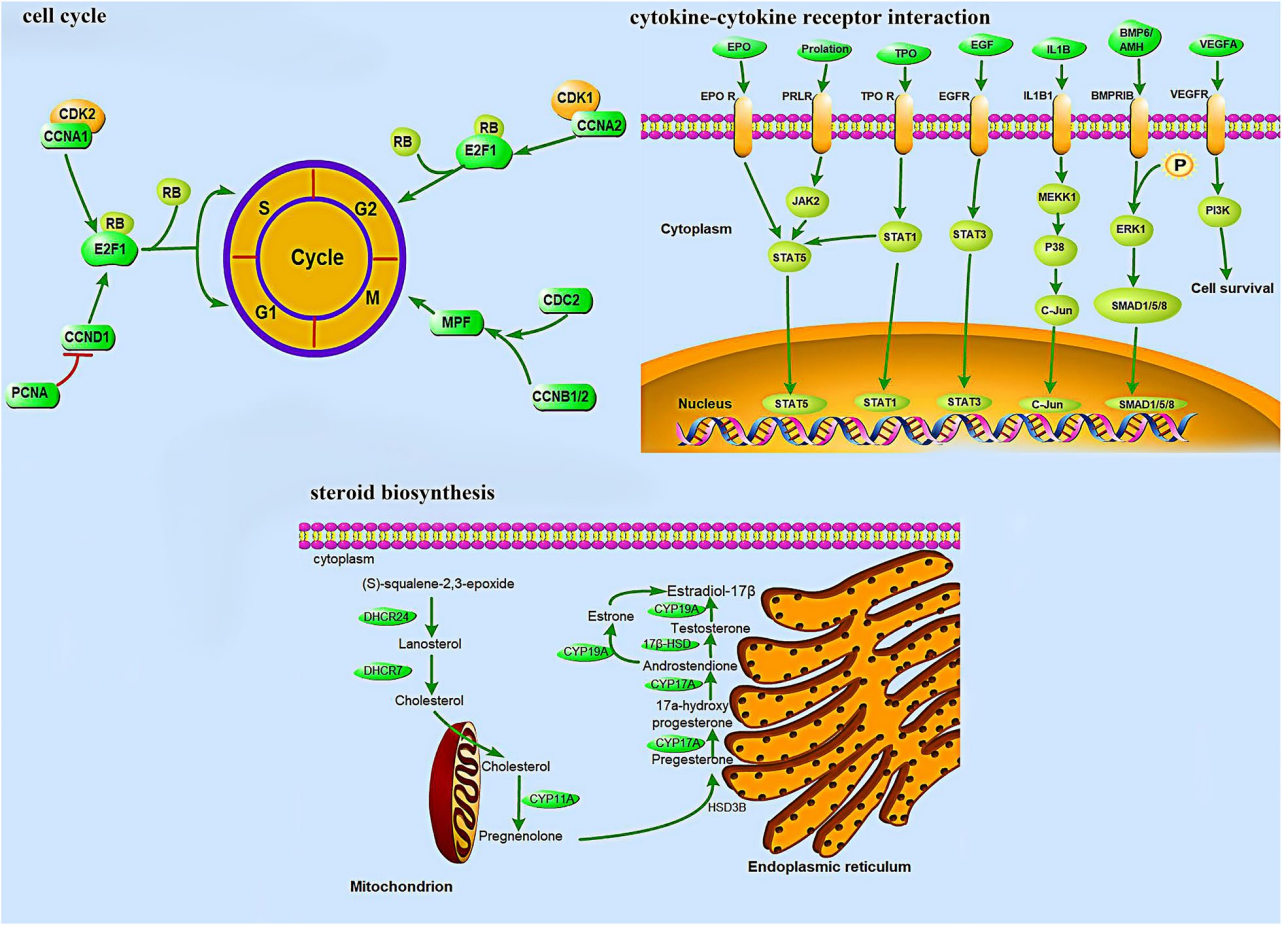
“Cytokine–cytokine receptor interaction”, “steroid biosynthesis” and “cell cycle” pathway and the identified regulator

Analysis of the KEGG pathway enrichment revealed that the 1413 DEGs participated in 242 pathways (Additional file 7), among which the largest category was “metabolic pathways” (ko01100, 11.18%), which had 158 annotated genes. Furthermore, 397 DEGs were significantly enriched (*p* ≤ 0.05) in 13 pathways, several of which were related to reproduction, such as “cytokine–cytokine receptor interaction”, “cell cycle”, and “steroid biosynthesis” (Figs. 4 and 5, Additional file 8: Figure S3, Additional file 9: Figure S4, Additional file 10: Figure S5 and Table 3). And these DEGs up- and down-regulated also shown in Fig. 5. The significantly enriched DEGs, including *PRLR*, *BMPR1B*, *EPOR*, kit Hardy (*KIT*), interleukin 1 beta (*IL1B*), cyclin A2 (*CCNA2*), cyclin B1 (*CCNB1*), and proliferating cell nuclear antigen (*PCNA*), have been reported to be involved in follicular development, hormone secretion, luteinization, and pregnancy maintenance.

**Table 3:** The significantly enriched pathways among the DEGs between the pregnant and nonpregnant

Pathway	DEGs with pathway annotation (1413)	All genes with pathway annotation (22,885)	*p* value	Q value	Pathway ID
Complement and coagulation cascades	51 (3.61%)	235 (1.03%)	2.05E−15	4.95E−13	ko04610
*Staphylococcus aureus* infection	33 (2.34%)	181 (0.79%)	1.88E−08	2.28E−06	ko05150
Hematopoietic cell lineage	30 (2.12%)	212 (0.93%)	1.87E−05	1.21E−03	ko04640
Bile secretion	28 (1.98%)	192 (0.84%)	2.00E−05	1.21E−03	ko04976
Cytokine–cytokine receptor interaction	46 (3.26%)	395 (1.73%)	2.70E−05	1.31E−03	ko04060
DNA replication	12 (0.85%)	51 (0.22%)	4.89E−05	1.97E−03	ko03030
Systemic lupus erythematosus	31 (2.19%)	241 (1.05%)	8.77E−05	3.03E−03	ko05322
Axon guidance	44 (3.11%)	403 (1.76%)	0.000178193	5.39E−03	ko04360
Pertussis	23 (1.63%)	177 (0.77%)	0.000587109	1.58E−02	ko05133
Cell cycle	31 (2.19%)	273 (1.19%)	0.000795974	1.93E−02	ko04110
Vitamin digestion and absorption	13 (0.92%)	78 (0.34%)	0.000920767	2.03E−02	ko04977
Steroid biosynthesis	10 (0.71%)	54 (0.24%)	0.001541861	3.11E−02	ko00100

## Discussion

We successfully identified the gene expression profiles of ovarian tissues from pregnant and nonpregnant AWGs using RNA-Seq (Quantification) and analyzed the gene expression differences between the two libraries. Furthermore, the GO enrichment and KEGG pathways for the DEGs were analyzed.

In our present study, 1724 genes were found to be significantly differentially expressed between the two libraries. Among them, a large number of DEGs were associated with prolificacy processes, follicular development, ovulation control, hormone secretion, and luteinization or pregnancy maintenance. For example, genes previously associated with reproduction, including *PRLR*, *LHR*, *STAR*, *SPP1*, *BMP6*, 3β-hydroxysteroid dehydrogenase estradiol (*3BHSD*), 17-beta-dehydrogenase 12 (*HSD17B12*), and *BMPR1B*, were found to be upregulated in pAWGs compared to nAWGs. The upregulated expression of *PRLR* mRNA during pregnancy may be associated with corpus luteum function for maintaining pregnancy [34]. *LHR* is a specific membrane receptor on the granulosa and theca cells that binds to luteinizing hormone (LH), resulting in androgen and progesterone production. *LHR* may impact follicular development from the primary follicle stage onwards [35]. *3BHSD* plays a crucial role in the biosynthesis of all classes of hormonal steroids [36]. *HSD17B12* plays an important role in female fertility [37]. *STAR*, which is the key rate-limiting enzyme in the production of steroid hormones, is involved in the regulation of steroid hormone biosynthesis and follicular development in the mammalian ovary [9]. *SPP1*, which is also known as *OPN* (osteopontin), is localized to luteal cells during the luteal phase of the estrous cycle and is involved in the development and regression of the corpus luteum [11]. Previous research has indicated that BMP6 may regulate ovary granulosa cell steroidogenesis, ovarian hormone secretion, follicular growth, and luteinization [38, 39]. A *BMPRIB* mutation may decrease the ability of BMP to inhibit differentiation, increase *INHBA* (inhibin beta A) mRNA expression in granular cells, and enhance the responsiveness of granule cells to gonadotropin [40]. *PGR*, *FSHR*, *CYP19A1*, *ESR2*, *INHA*, *NHBA*, estradiol 17-beta-dehydrogenase 1 (*HSD17B1*), and inhibin beta B (*INHBB*) were found to be downregulated in pAWG. Recent studies showed that *PGR, ESR2, FSHR, CYP19A1, HSD17B1, INHA, INHBA* and *INHBB* played a very important role in hormone secretion, follicle growth, cell proliferation and apoptosis [41–43].

Our data showed some uniquely transcribed genes in nAWGs and pAWGs. Among these specifically expressed genes, two genes (*DEFB112* and *IFNE*) from nAWGs and two genes (*SST* and *SSTR1*) from pAWGs may be involved in related events during pregnancy. *DEFB112* is a type of B-defensin that is preferentially expressed in the male reproductive system and actively involved in sperm maturation and capacitation but is poorly documented. Further study is required to determine whether *DEFB112* is involved in the reproductive process in female animals [44]. *IFNE* is a novel type I *IFN* that has been detected in the lung, reproductive tissue, intestine and so on. *IFNE* may protect the female reproductive tract from viral and bacterial infection. Its expression was found to be at the lowest levels during diestrus and the highest levels at estrus [45]. *IFNE* can activate the JAK-STAT signaling pathway, which plays a very important role in reproduction [46]. *SST* is a 14-amino acid polypeptide with a short half-life that may inhibit the secretion of various hormones including GH and LH. *SSTR1* is somatostatin receptor subtype 1. *SST* and its receptors are present in the ovary in various species. It was reported that *SSTR2* and *5* activation modulate ovarian steroidogenesis by upregulating endogenous BMP activity in growing follicles [47]. Previous studies have shown that *SSTR1* inhibits cAMP accumulation by coupling to pertussis toxin-sensitive G proteins [48]. However, the specific functions of *SSTR1* are unclear and require further study.

Regarding GO enrichment analysis, we found that some of the correlated genes were enriched into reproduction-related GO terms. For example, *CYP19A1*, *ESR2*, *PGR INHA*, *INHBA* and *STAR* were enriched into GO terms for reproduction, reproduction process, molecular transducer activity, and transporter activity. The results indicated that these genes may play a specific role in goat reproduction. For the uniquely transcribed genes, the two genes (*ENC1* and *RGS17*) from nAWGs were enriched for signaling, enzyme regulator activity, and molecular transducer activity GO terms, while the six genes (*DCDC2*, *TBC1D15*, *PTHRP*, *SLC1A2*, *RDH16* and *SST*) from pAWGs were enriched for biological regulation, enzyme regulator activity, growth, metabolic process, and response to stimulus GO terms. Our data indicated that these genes may be related to cell proliferation and hormone secretion.

Three pathways, including “cytokine–cytokine receptor interaction”, “steroid biosynthesis” and “cell cycle”, were selected from the 13 significantly enriched pathways and were found to be intimately associated with mammalian reproduction. Most of the DEGs participating in cytokine–cytokine receptor interaction and steroid biosynthesis pathways were upregulated, including *EPOR*, *KIT*, *IL1B*, *PRLR* and *BMPR1B*, indicating that these pathways are activated in the ovaries of pregnant goats. It was shown that *EPOR* and *IL1B* might play a role in establishing pregnancy [49, 50]. *KIT* plays a very important role in the maintenance of the primordial follicle reserve and in primary to secondary follicle transition [51]. Most DEGs were downregulated in the cell cycle pathway, such as *CCNA2*, *CCNB1* and so on, and only one gene (*cyclin D1*, *CCND1*) was unregulated. *CCNA2* regulates cell cycle progression by interacting with *CDK* kinases and is involved in the G2/M transition. *CCNB1* is a regulatory protein involved in mitosis. Research indicates that the downregulated expression of *CCNA2* may promote primordial follicle activation, while *CCNB1* plays a significant role in promoting the maturation of large-follicle oocytes [52, 53]. *CCND1* is a protein required for progression through G1 phase of the cell cycle and its main function is to promote cell proliferation [54]. It can be inferred that these genes may be involved in cell cycle regulation, granulosa cell proliferation and follicular development.

Furthermore, some other signaling pathways associated with reproduction were also identified in our current analysis. These pathways included the ECM-receptor interaction, focal adhesion, and Jak-STAT, Wnt, insulin, and p53 signaling pathways, suggesting that some molecules in these pathways might also be involved in goat reproduction or maintaining the physiological activity of the ovary. However, the exact relationship between these signaling networks is not fully understood and further research is needed. Generally, the current KEGG pathway analyses have provided a solid basis for future work.

## Conclusion

In conclusion, this study presents the transcriptomic profiles of ovarian tissue from pregnant and nonpregnant AWGs using RNA-Seq technology. 1724 genes were detected to be significantly differentially expressed in pAWGs compared with nAWGs. Among them, 1033 genes were upregulated and 691 were downregulated in pAWGs, suggesting that DEGs may play an important role in the regulation of goat ovarian function. GO annotation and KEGG pathway analyses were conducted on the DEGs between the two libraries, and 13 pathways were pointed out for significantly enriched, in which “cytokine–cytokine receptor interaction”, “cell cycle”, and “steroid biosynthesis” were found to be associated with reproduction. The DEGs and pathways identified could facilitate further to elucidate the functions of DEGs in goat follicular development and hormone secretion, which will help us to understand the relationship between genes and mammalian reproduction and provide a solid foundation for future studies.

## Additional files

**Additional file 1.** Summary of the sequenced reads mapped to the *Capra hircus* reference genes and genome.

**Additional file 2.** All detected genes in the two libraries.

**Additional file 3.** Gene coverage distribution in the two ovary libraries.

**Additional file 4.** plot indicating the log-transformed gene expression levels and DEG distributions between the pregnant and nonpregnant samples.

**Additional file 5.** Differential gene detected in two libraries.

**Additional file 6.** Gene Ontology (GO) classifications of two libraries.

**Additional file 7.** KEGG pathway analysis of two libraries.

**Additional file 8.** Cytokine–cytokine receptor interaction signal pathway.

**Additional file 9.** Steroid biosynthesis signal pathway.

**Additional file 10.** Cell cycle pathway.

## References

[1] Ling YH, Ren CH, Guo XF, Xu LN, Huang YF, Luo JC (2014). Identification and characterization of microRNAs in the ovaries of multiple and uniparous goats (*Capra hircus*) during follicular phase. BMC Genomics..

[2] Chen S, Cheng GL, Zhu DJ, Jiang XC, Zhao HL (2009). Determination on the body properties and meat performance of Anhui white goat. Anim Husb Feed Sci..

[3] Gram A, Boos A, Kowalewski MP (2014). Uterine and placental expression of canine oxytocin receptor during pregnancy and normal and induced parturition. Reprod Domest Anim.

[4] Agaoglu OK, Agaoglu AR, Guzeloglu A, Kurar E, Kayis SA, Ozmen O (2015). Expression of hypoxia-inducible factors and vascular endothelial growth factor during pregnancy in the feline uterus. Theriogenology.

[5] Stephens SM, Moley KH (2009). Follicular origins of modern reproductive endocrinology. Am J Physiol Endocrinol Metab.

[6] Ling YH, Guo XF, Chen T, Ding JP, Ma YH, Chu MX (2016). Characterization and analysis of differentially expressed microRNAs in hircine ovaries during the follicular and luteal phases. Anim Reprod Sci..

[7] Orisaka M, Jiang JY, Orisaka S, Kotsuji F, Tsang BK (2009). Growth differentiation factor 9 promotes rat preantral follicle growth by up-regulating follicular androgen biosynthesis. Endocrinology.

[8] Sargent KM, Lu N, Clopton DT, Pohlmeier WE, Brauer VM, Ferrara N (2015). Loss of vascular endothelial growth factor A (VEGFA) isoforms in granulosa cells using pDmrt-1-Cre or Amhr2-Cre reduces fertility by arresting follicular development and by reducing litter size in female mice. PLoS ONE.

[9] Yamashita H, Murayama C, Takasugi R, Miyamoto A, Shimizu T (2011). BMP-4 suppresses progesterone production by inhibiting histone H3 acetylation of StAR in bovine granulosa cells in vitro. Mol Cell Biochem.

[10] Galloway SM, McNatty KP, Cambridge LM, Laitinen MP, Juengel JL, McLaren RJ (2000). Mutations in an oocyte-derived growth factor gene (BMP15) cause increased ovulation rate and infertility in a dosage-sensitive manner. Nat Genet.

[11] Poole DH, Ndiaye K, Pate JL (2013). Expression and regulation of secreted phosphoprotein 1 in the bovine corpus luteum and effects on T cell lymphocyte chemotaxis. Reproduction.

[12] Piccinni MP, Maggi E, Romagnani S (2000). Role of hormone-controlled T-cell cytokines in the maintenance of pregnancy. Biochem Soc Trans.

[13] Saraiva MV, Celestino JJ, Araújo VR, Chaves RN, Almeida AP, Lima-Verde IB (2011). Expression of follicle-stimulating hormone receptor (FSHR) in goat ovarian follicles and the impact of sequential culture medium on in vitro development of caprine preantral follicles. Zygote..

[14] Magalhães-Padilha DM, Duarte ABG, Araújo VR, Saraiva MVA, Almeida AP, Rodrigues GQ (2012). Steady-state level of insulin-like growth factor-I (IGF-I) receptor mRNA and the effect of IGF-I on the in vitro culture of caprine preantral follicles. Theriogenology.

[15] Frota IM, Leitão CC, Costa JJ, van den Hurk R, Saraiva MV, Figueiredo JR (2013). Levels of BMP-6 mRNA in goat ovarian follicles and in vitro effects of BMP-6 on secondary follicle development. Zygote..

[16] Pramod RK, Sharma SK, Singhi A, Pan S, Mitra A (2013). Differential ovarian morphometry and follicular expression of BMP15, GDF9 and BMPR1B influence the prolificacy in goat. Reprod Domest Anim.

[17] An XP, Hou JX, Li G, Peng JY, Liu XQ, Liu HY (2012). Analysis of differentially expressed genes in ovaries of polytocous versus monotocous dairy goats using suppressive subtractive hybridization. Reprod Domest Anim.

[18] Magalhães-Padilha DM, Geisler-Lee J, Wischral A, Gastal MO, Fonseca GR, Eloy YR (2013). Gene expression during early folliculogenesis in goats using microarray analysis. Biol Reprod.

[19] Ling YH, Xiang H, Li YS, Liu Y, Zhang YH, Zhang ZJ (2014). Exploring differentially expressed genes in the ovaries of uniparous and multiparous goats using the RNA-Seq (Quantification) method. Gene.

[20] Mortazavi A, Williams BA, McCue K, Schaeffer L, Wold B (2008). Mapping and quantifying mammalian transcriptomes by RNA-Seq. Nat Methods.

[21] Gunawan A, Sahadevan S, Neuhoff C, Große-Brinkhaus C, Gad A, Frieden L (2013). RNA deep sequencing reveals novel candidate genes and polymorphisms in boar testis and liver tissues with divergent androstenone levels. PLoS ONE.

[22] Huang W, Khatib H (2010). Comparison of transcriptomic landscapes of bovine embryos using RNA-Seq. BMC Genomics..

[23] Hackett NR, Butler MW, Shaykhiev R, Salit J, Omberg L, Rodriguez-Flores JL (2012). RNA-Seq quantification of the human small airway epithelium transcriptome. BMC Genomics..

[24] Chen HY, Shen H, Jia B, Zhang YS, Wang XH, Zeng XC (2015). Differential gene expression in ovaries of Qira Black sheep and Hetian sheep using RNA-Seq technique. PLoS ONE.

[25] Miao X, Luo Q, Qin X (2016). Genome-wide transcriptome analysis in the ovaries of two goats identifies differentially expressed genes related to fecundity. Gene.

[26] Kerpedjiev P, Frellsen J, Lindgreen S, Krogh A (2014). Adaptable probabilistic mapping of short reads using position specific scoring matrices. BMC Bioinform.

[27] Li R, Yu C, Li Y, Lam TW, Yiu SM, Kristiansen K (2009). SOAP2: an improved ultrafast tool for short read alignment. Bioinformatics.

[28] Audic S, Claverie JM (1997). The significance of digital gene expression profiles. Genome Res.

[29] Wang L, Feng Z, Wang X, Wang X, Zhang X (2010). DEGseq: an R package for identifying differentially expressed genes from RNA-Seq data. Bioinformatics.

[30] Ishida-Takagishi M, Enomoto A, Asai N, Ushida K, Watanabe T, Hashimoto T (2012). The Dishevelled-associating protein Daple controls thenon-canonical Wnt/Rac pathway and cell motility. Nat Commun..

[31] Conesa A, Götz S, García-Gómez JM, Terol J, Talón M, Robles M (2005). Blast2GO: a universal tool for annotation, visualization and analysis in functional genomics research. Bioinformatics.

[32] Ye J, Fang L, Zheng H, Zhang Y, Chen J, Zhang Z (2006). WEGO: a web tool for plotting GO annotations. Nucleic Acids Res.

[33] Kanehisa M, Araki M, Goto S, Hattori M, Hirakawa M, Itoh M (2008). KEGG for linking genomes to life and the environment. Nucleic Acids Res.

[34] Zi XD, Chen DW, Wang HM (2012). Molecular characterization, mRNA expression of prolactin receptor (PRLR) gene during pregnancy, nonpregnancy in the yak (*Bos grunniens*). Gen Comp Endocrinol.

[35] Phoophitphong D, Srisuwatanasagul S, Tummaruk P (2017). Immunohistochemical localization of luteinizing hormone receptor in the cyclic gilt ovary. Anat Histol Embryol.

[36] Lachance Y, Luu-The V, Labrie C, Simard J, Dumont M, de Launoit Y (1990). Characterization of human 3 beta-hydroxysteroid dehydrogenase/delta 5-delta 4-isomerase gene and its expression in mammalian cell. J Biol Chem.

[37] Kemiläinen H, Adam M, Mäki-Jouppila J, Damdimopoulou P, Damdimopoulos AE, Kere J (2016). The hydroxysteroid (17β) dehydrogenase family Gene HSD17B12 is involved in the prostaglandin synthesis pathway, the ovarian function, and regulation of fertility. Endocrinology.

[38] Campbell BK, Kendall NR, Baird DT (2009). Effect of direct ovarian infusion of bone morphogenetic protein 6 (BMP6) on ovarian function in sheep. Biol Reprod.

[39] Khalaf M, Morera J, Bourret A, Reznik Y, Denoual C, Herlicoviez M (2013). BMP system expression in GCs from polycystic ovary syndrome women and the in vitro effects of BMP4, BMP6, and BMP7 on GC steroidogenesis. Eur J Endocrinol.

[40] Fabre S, Pierre A, Pisselet C, Mulsant P, Lecerf F, Pohl J (2003). The Booroola mutation in sheep is associated with an alteration of the bone morphogenetic protein receptor-IB functionality. J Endocrinol.

[41] Komatsu K, Masubuchi S (2017). The concentration-dependent effect of progesterone on follicle growth in the mouse ovary. J Reprod Dev..

[42] Chakravarthi VP, Khristi V, Ghosh S, Yerrathota S, Dai E, Roby KF (2018). ESR2 Is essential for gonadotropin-induced Kiss1 expression in granulosa cells. Endocrinology.

[43] Cadoret V, Frapsauce C, Jarrier P, Maillard V, Bonnet A, Locatelli Y (2017). Molecular evidence that follicle development is accelerated in vitro compared to in vivo. Reproduction.

[44] Patil AA, Cai Y, Sang Y, Blecha F, Zhang G (2005). Cross-species analysis of the mammalian beta-defensin gene family: presence of syntenic gene clusters and preferential expression in the male reproductive tract. Physiol Genomics.

[45] Fung KY, Mangan NE, Cumming H, Horvat JC, Mayall JR, Stifter SA (2013). Interferon-ε protects the female reproductive tract from viral and bacterial infection. Science.

[46] Yang L, Xu L, Li Y, Li J, Bi Y, Liu W (2013). Molecular and functional characterization of canine interferon-epsilon. J Interferon Cytokine Res.

[47] Nakamura E, Otsuka F, Inagaki K, Tsukamoto N, Ogura-Ochi K, Miyoshi T (2013). Involvement of bone morphogenetic protein activity in somatostatin actions on ovarian steroidogenesis. J Steroid Biochem Mol Biol.

[48] Hadcock JR, Strnad J, Eppler CM (1994). Rat somatostatin receptor type 1 couples to G proteins and inhibition of cyclic AMP accumulation. Mol Pharmacol.

[49] Geisert R, Fazleabas A, Lucy M, Mathew D (2012). Interaction of the conceptus and endometrium to establish pregnancy in mammals: role of interleukin 1β. Cell Tissue Res.

[50] Ji YQ, Zhang YQ, Li MQ, Du MR, Wei DD, Li DJ (2011). EPO improves the proliferation and inhibits apoptosis of trophoblast and decidual stromal cells through activating STAT-5 and inactivating p38 signal in human early pregnancy. Int J Clin Exp Pathol..

[51] John GB, Shidler MJ, Besmer P, Castrillon DH (2009). Kit signaling via PI3K promotes ovarian follicle maturation but is dispensable for primordial follicle activation. Dev Biol..

[52] Tong Y, Li F, Lu Y, Cao Y, Gao J, Liu J (2013). Rapamycin-sensitive mTORC1 signaling is involved in physiological primordial follicle activation in mouse ovary. Mol Reprod Dev.

[53] Liu HL, Gao Y, Zhai B, Jiang H, Ding Y, Zhang L (2016). The Effects of polyadenylation status on MPFs during in vitro porcine oocyte maturation. Cell Physiol Biochem.

[54] Fu M, Wang C, Li Z, Sakamaki T, Pestell RG (2004). Minireview: cyclin D1: normal and abnormal functions. Endocrinology.

