# Supplementary figures and images for: Comparative analysis of differentially expressed genes between the ovaries from pregnant and nonpregnant goats using RNA-Seq
Source: J Biol Res (Thessalon). 2019 May 6;26:3. doi: 10.1186/s40709-019-0095-9 (PMC6503366; doi:10.1186/s40709-019-0095-9)

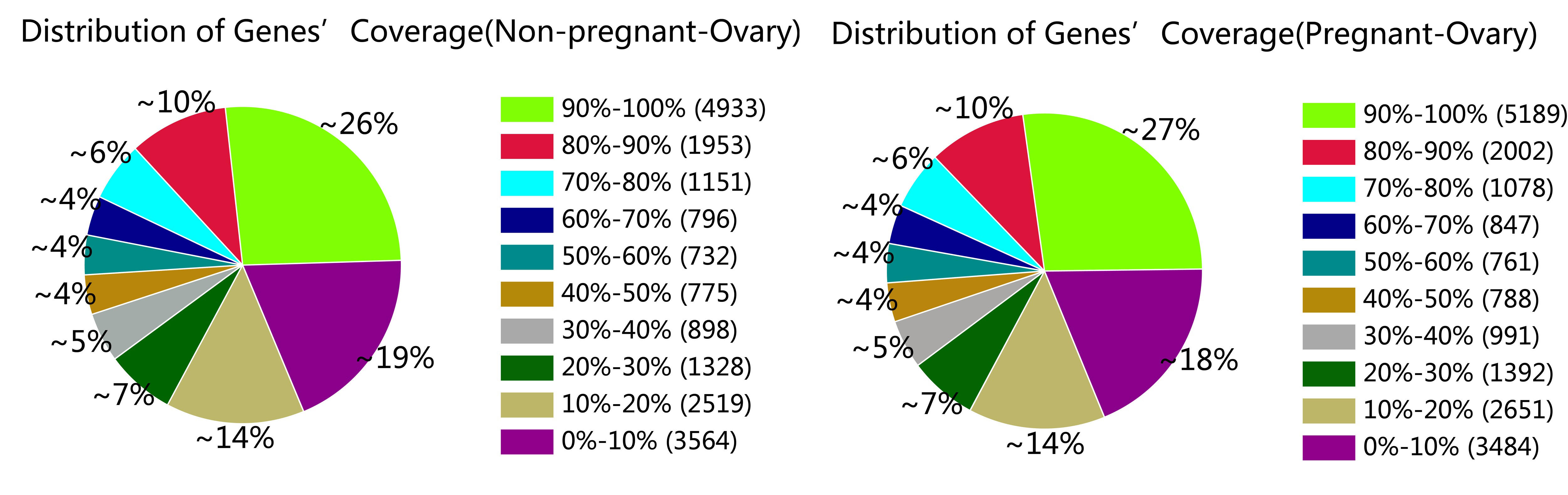

Supplement: Supplementary file 3 — Additional file 3. Gene coverage distribution in the two ovary libraries. [file 40709_2019_95_MOESM3_ESM.tif]

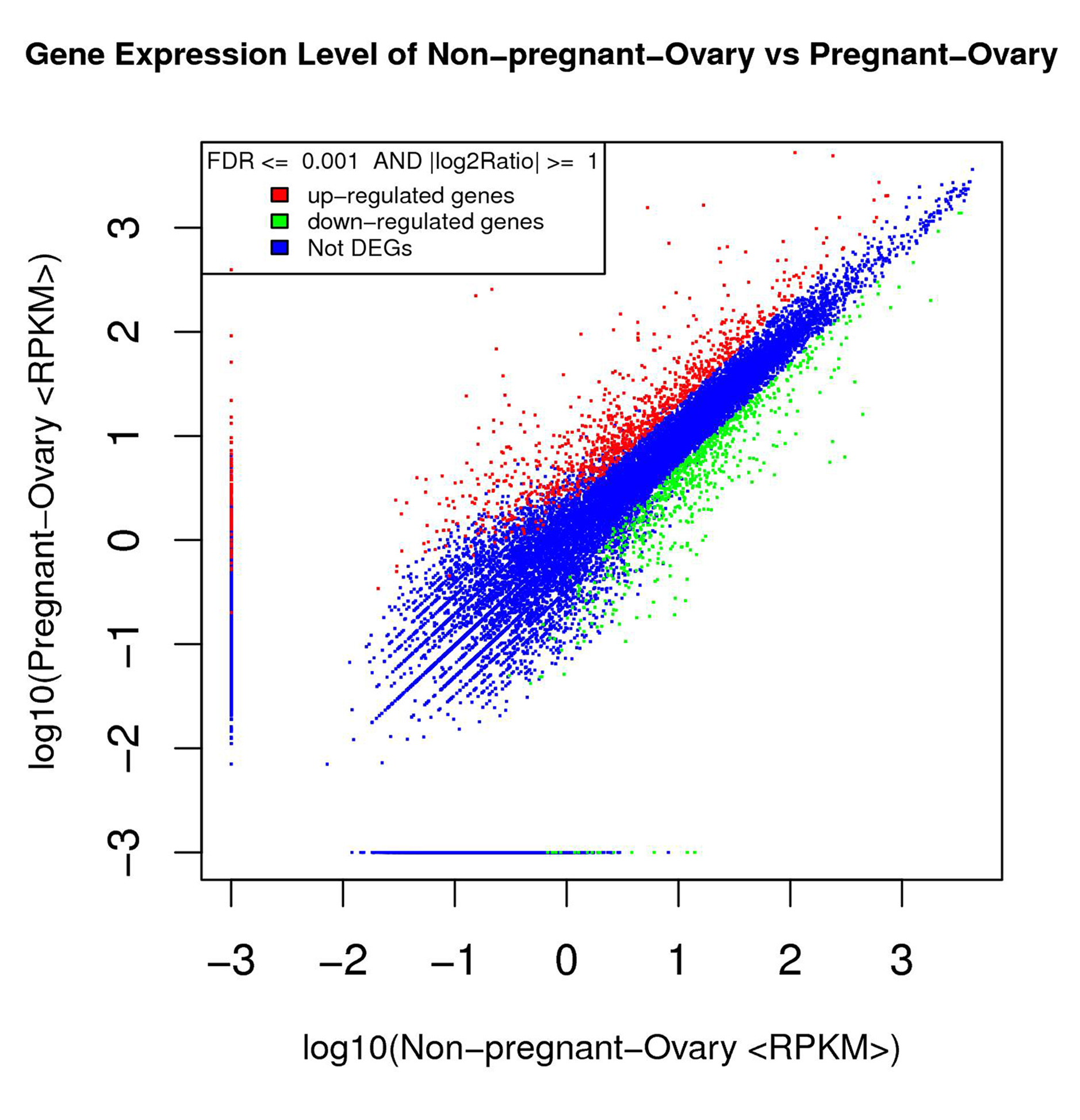

Supplement: Supplementary file 4 — Additional file 4. plot indicating the log-transformed gene expression levels and DEG distributions between the pregnant and nonpregnant samples. [file 40709_2019_95_MOESM4_ESM.tif]

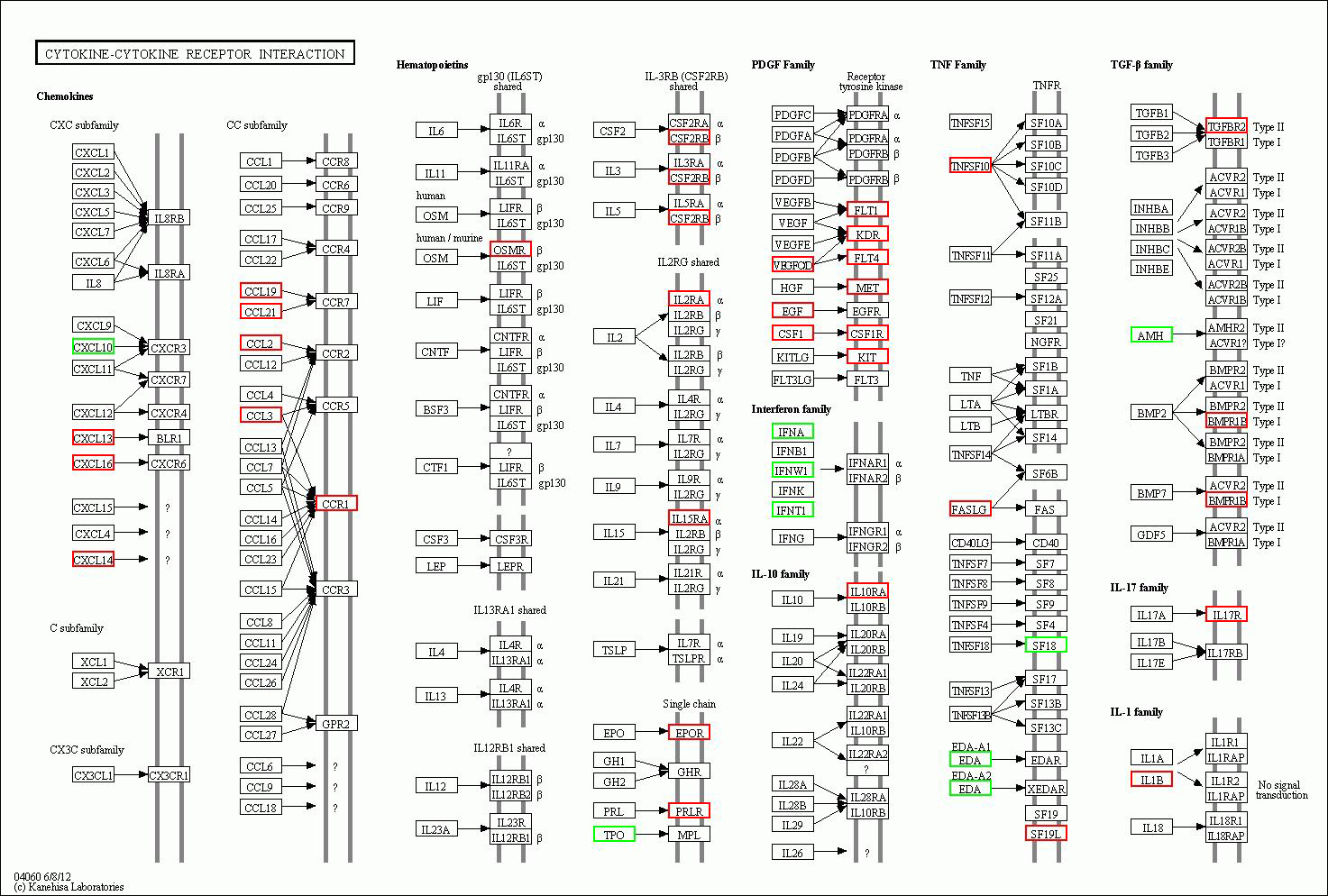

Supplement: Supplementary file 8 — Additional file 8. Cytokine–cytokine receptor interaction signal pathway. [file 40709_2019_95_MOESM8_ESM.tif]

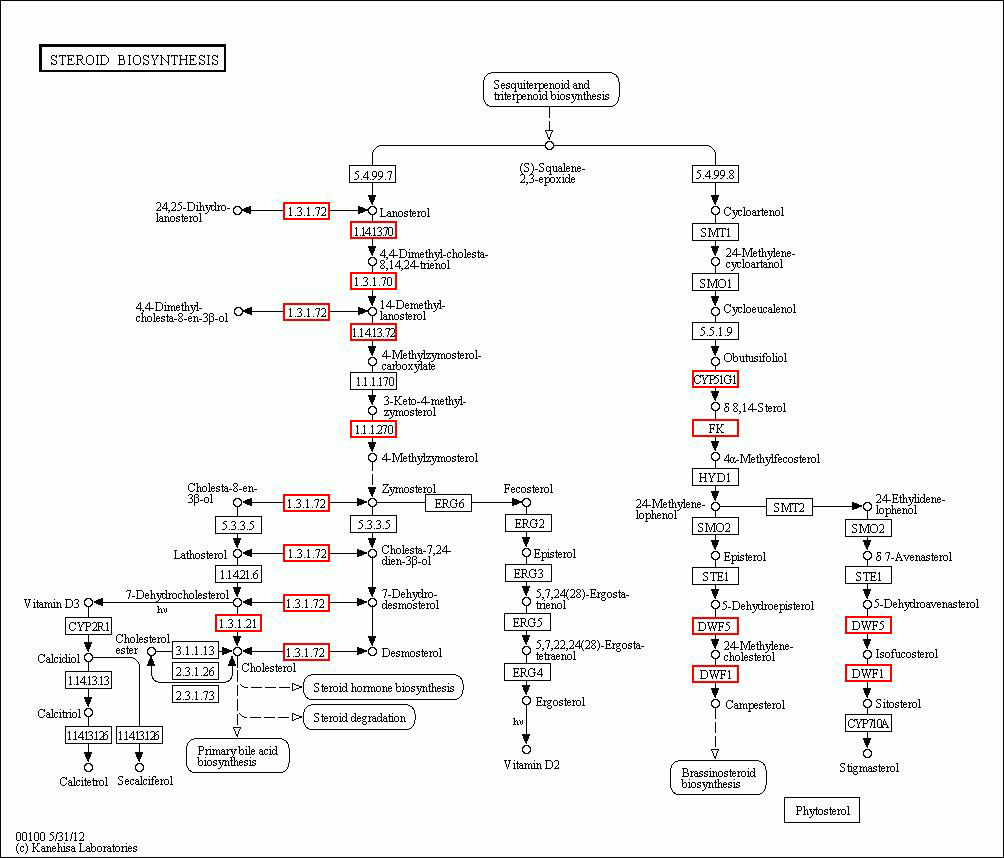

Supplement: Supplementary file 9 — Additional file 9. Steroid biosynthesis signal pathway. [file 40709_2019_95_MOESM9_ESM.tif]

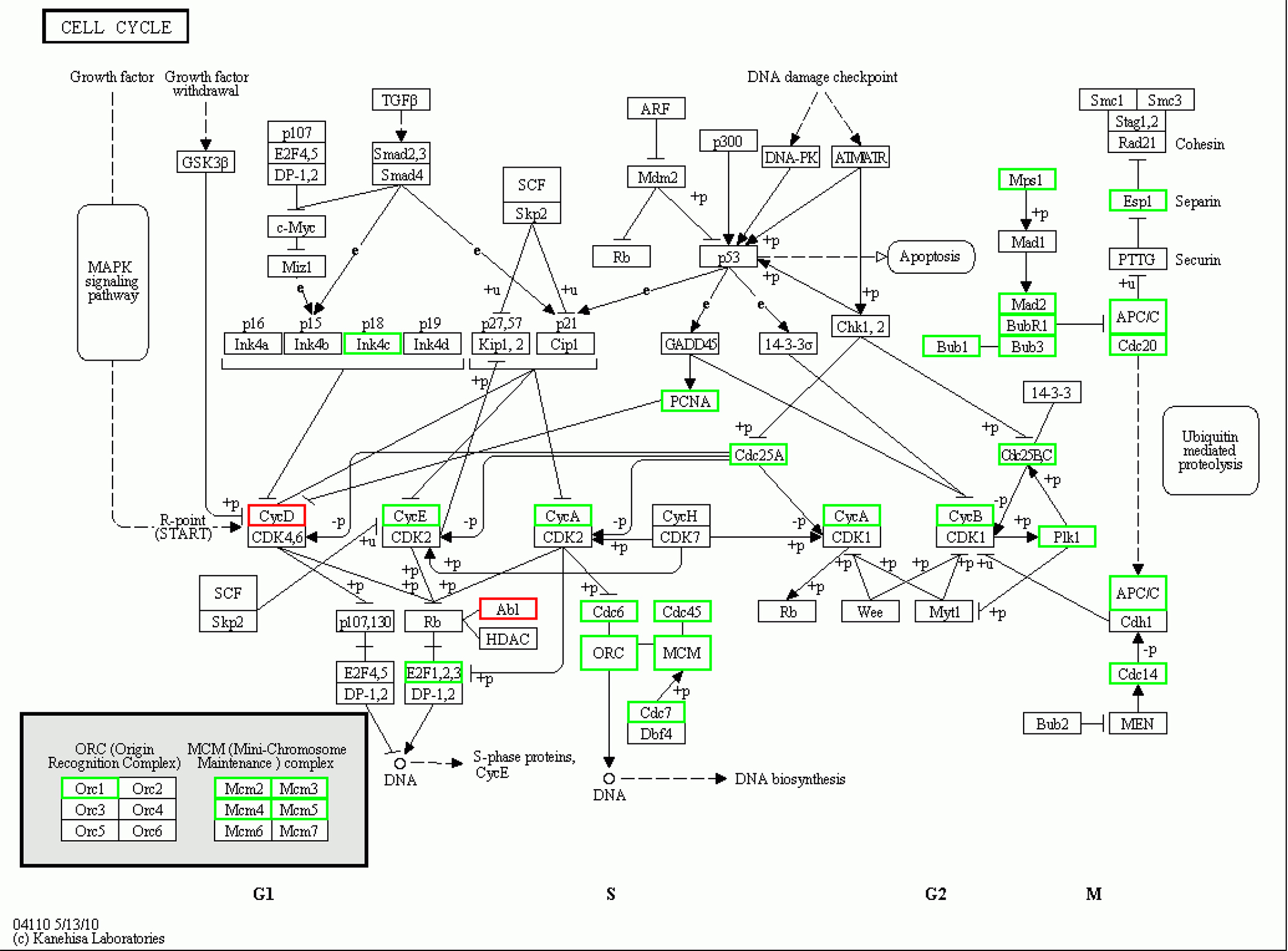

Supplement: Supplementary file 10 — Additional file 10. Cell cycle pathway. [file 40709_2019_95_MOESM10_ESM.tif]
